# Autoimmune Encephalitis in Late-Onset Seizures: When to Suspect and How to Treat

**DOI:** 10.3389/fneur.2021.633999

**Published:** 2021-04-07

**Authors:** Marie Süße, Maria Zank, Viola von Podewils, Felix von Podewils

**Affiliations:** Department of Neurology, Epilepsy Center, University Medicine Greifswald, Greifswald, Germany

**Keywords:** autoimmune encephalitis, late onset seizures, neural antibodies, epilepsy of unknown origin, outcome

## Abstract

**Objective:** This study was conducted to elucidate prevalence, clinical features, outcomes, and best treatment in patients with late-onset seizures due to autoimmune encephalitis (AE).

**Methods:** This is a single-institution prospective cohort study (2012–2019) conducted at the Epilepsy Center at the University of Greifswald, Germany. A total of 225 patients aged ≥50 years with epileptic seizures were enrolled and underwent an MRI/CT scan, profiling of neural antibodies (AB) in serum and cerebrospinal fluid (CSF), and neuropsychological testing. On the basis of their work-up, patients were categorized into the following three cohorts: definite, suspected, or no AE. Patients with definite and suspected AE were subsequently treated with immunosuppressive therapy (IT) and/or anti-seizure drug (ASD) therapy and were followed up (FU) regarding clinical and seizure outcome.

**Results:** Of the 225 patients, 17 (8%) fulfilled the criteria for definite or suspected AE according to their AB profile and MRI results. Compared with patients with no evidence of AE, those with AE were younger (*p* = 0.028), had mesial temporal neuropsychological deficits (*p* = 0.001), frequently had an active or known malignancy (*p* = 0.006) and/or a pleocytosis (*p* = 0.0002), and/or had oligoclonal bands in CSF (*p* = 0.001). All patients with follow-up became seizure-free with at least one ASD. The Modified Rankin scale (mRS) at hospital admission was low for patients with AE (71% with mRS ≤2) and further decreased to 60% with mRS ≤2 at last FU.

**Significance:** AE is an important etiology in late-onset seizures, and seizures may be the first symptom of AE. Outcome in non-paraneoplastic AE was favorable with ASD and IT. AB testing in CSF and sera, cerebral MRI, CSF analysis, and neuropsychological testing for mesial temporal deficits should be part of the diagnostic protocol for AE following late-onset seizures.

## Introduction

Epilepsy and new onset seizures in elderly patients are an important health issue of the aging population (1, 2). Epileptic seizures are a core symptom of autoimmune encephalitis (AE) (3, 4), and autoimmune epilepsy has been reported to account for up to 20% of epilepsy of unknown etiology (5). The prevalence ranges from 6% up to 37% (3, 6), with non-paraneoplastic AE being more common than paraneoplastic AE. The diagnostic criteria for definite autoimmune limbic encephalitis proposed by Graus et al. (4) enable the diagnosis of AE even in the absence of neural antibodies and include neuropsychiatric symptoms of new-onset seizures, bilateral changes in magnetic resonance imaging (MRI), such as T2/fluid-attenuated inversion recovery (FLAIR) hyperintense signal alterations prominently in the medial temporal lobes, and either cerebrospinal fluid (CSF) pleocytosis or EEG abnormalities (4). Diagnostic criteria for possible AE include subacute neuropsychiatric deficits and either new focal CNS findings, seizures, CSF pleocytosis, or pathognomonic MRI features (4). The diagnostic criteria for neural antibody (AB) negative but probable AE include the detection of oligoclonal bands (OCB) in CSF (4). Small case series suggest using FDG-PET in cases of suspected AE and normal MRI, as FDG-PET has been reported to be more sensitive than MRI (7–9).

The presence of neural antibodies (AB) in serum and/or CSF can be suggestive of an autoimmune origin, but their absence does not exclude autoimmunity (10). Furthermore, their positivity is not always pathognomonic for AE (11), as described for low-titer anti-CASPR2 AB, VGKC AB not reactive with LGI1/CASPR2, AB against mouse neuropil, and low-titer GAD AB in serum (12–14). Since AE is a potentially treatable etiology of epileptic seizures (11), its identification in the diagnostic work-up of epileptic seizures is crucial (15). The application of the above-mentioned criteria proposed by Graus et al. (4) can be misleading in routine clinical practice (11, 16). For example, in the elderly, AB-associated central nervous system (CNS) syndromes often present without characteristic features of inflammation, such as pleocytosis in CSF or corresponding MRI changes, especially in patients aged 60 years or older (17). Furthermore, isolated seizures can precede other features of AE (17), thus complicating a timely recognition of possible AE (18).

Therefore, the aim of this study was to investigate the prevalence of AE in a large cohort of patients with late-onset seizures and to find predictors of when to suspect AE in late-onset seizures. We furthermore aimed to characterize the long-term outcomes and provide clinicians with recommendations for an adequate treatment in this challenging group of patients.

## Methods and Materials

In this prospective single-institution cohort study, patients from the general neurology department, the neurological intensive care unit, and the epilepsy monitoring unit who underwent diagnostic workup after epileptic seizures including CSF analysis between 2012 and 2019 were studied at the University Hospital of Greifswald, Germany (serving a population of about 500,000 people).

### Patient Cohort

Patients ≥50 years of age with status epilepticus, repetitive seizures for ≤6 months, or one single seizure (further referred to as seizure disorder) and CSF analysis were included. Cerebrospinal fluid analysis was performed in all patients with a first manifestation of a seizure disorder. Cerebrospinal fluid analysis after a first seizure disorder is a clinical standard in our institution, especially in patients aged 50 years or older, with certain exceptions, such as clear evidence of a generalized genetic epilepsy syndrome. All patients without CSF analysis regardless of the reason (for example, those who have refused consent or have oral anticoagulation) were excluded from the study. Patients with suspected infectious etiology of the seizure disorder based on CSF analysis (cell count, lactate, and protein elevation in CSF analysis and/or evidence of viral or bacterial infection in microbiological analysis) without further evidence of a comorbid autoimmune encephalitis were also excluded from the study. Seizures were classified according to the International League Against Epilepsy (ILAE) classification of seizure types (19). All patients underwent MRI or CT imaging (if MRI was not possible), neuropsychological testing if feasible, and CSF analysis as part of the study protocol.

Immunosuppressive therapy (IT) and/or anti-seizure drug (ASD) treatment was started at the discretion of the treating physician. First-line IT included prednisolone (per os/intravenous), plasma exchange (PEX), immunoabsorption (IA), or intravenous immunoglobulins, second-line IT included further immunosuppressive therapy (rituximab, methotrexate, cyclophosphamide, azathioprine). Tumor screening, including CT scan of the thorax/abdomen and gynecological/urological investigations, was performed in cases of suspected paraneoplastic AE in 6-month intervals for at least 2 years.

Patients were categorized in three cohorts and defined as follows: (1) definite autoimmune encephalitis (dAE): neural AB positivity and/or bilateral hyperintense signals in T2-FLAIR MRI sequences either restricted to the medial temporal lobes or in multifocal areas compatible with demyelination or inflammation (4); (2) suspected AE (sAE): positive neural AB at either borderline titers in serum or non-specific neuropil AB and/or unilateral mesial temporal MRI T2-FLAIR hyperintense signal alterations; additional findings consistent with AE may support the diagnosis, such as neuropsychological findings compatible with AE and/or detection of an elevated cell count in CSF or OCB in CSF and/or EEG epileptic activity or regional slowing involving the temporal lobes; and (3) no proof of AB and no signs for AE on brain imaging (AE negative, nAE).

Follow-up (FU) investigation was not part of the initial study protocol and was carried out in 2019 after the end of the intended observation period. Follow-up included clinical and seizure outcomes in cohorts 1 and 2 (dAE/sAE) if available and was assessed with a structured telephone interview or data evaluation from the available medical charts. Minimum follow-up time was 6 months.

### Neuropsychological Assessment

Standardized neuropsychological assessment was feasible in 11 out of 17 (65%) dAE/sAE patients (*n* = 6 for dAE and *n* = 5 for sAE). Test protocols focused mainly on attention and cognitive speed as well as executive, language, and memory functions. These were either assessed in seven patients using CERAD-plus (Consortium to Establish a Registry for Alzheimer's Disease) or using a comprehensive test battery (*n* = 4 patients) including the following tests: MWT-B (Mehrfachwahlwortschatztest, verbal intelligence) for an estimated premorbid performance level, BVMT-R (Brief Visual Memory Test-Revised) or ROCFT (Rey-Osterrieth Complex Figure Test) for figural memory, CVLT (California Verbal Learning Test) or VLMT (Verbal Learning and Memory Test, the German equivalent of the Rey Auditory Verbal Learning Test) for verbal memory, TAP (Testbatterie zur Aufmerksamkeitsprüfung), digit span (WAIS IV, Wechsler Adult Intelligence Test), TMT (Trail Making Test) or Stroop Test for attention and executive functions, and phonemic and/or semantic verbal fluency (Regensburger Wortschatz-Test) and naming for language functions (see Supplementary Tables 1, 2). Test performance of >1 SD below the mean was defined as a cognitive deficit. Neuropsychological test performance was interpreted by certified neuropsychologists. A focus of impairments on verbal and/or figural memory (especially delayed recall and recognition) was defined as a mesial temporal deficit, consistent with AE.

### Laboratory Analysis

Laboratory analyses were performed in the Interdisciplinary CSF laboratory of the University Medicine Greifswald. Laboratory analyses were performed as described previously (20). In brief, cell counts were determined microscopically using a Fuchs-Rosenthal counting chamber. The calculation of intrathecal IgG was performed according to Reiber's formula (21). OCB were determined by isoelectric focusing with a semiautomated agarose electrophoresis system (Hydragel 9 CSF, Sebia Hydrasys 2Scan, Sebia GmbG, Fulda Germany). OCB positivity was defined with ≥2 isolated bands in CSF.

The determination of neural AB in CSF and serum was performed by the MVZ Labor Krone GbR, Siemensstraße 40, 32105 Bad Salzuflen, Germany (for details see Supplementary Material). All patients were tested for serum and CSF AB against GAD65, NMDAR, GABA(B)R, IGLON5, AMPA1/2, DPPX, LGI1, CASPR2, GlyRs, mGluR5, mGluR1, and atypical AB against neuropil as well as AB against Amphiphysin, CV2/CRMP5, Ma2/Ta, Ri, Yo, Hu, Recoverin, Sox1, Titin, Zic4, and DNER/Tr.

### Statistical Analysis

Statistical analysis was performed using SPSS 23.0 (IBM Co., Armonk, New York, USA). Kolmogorov-Smirnov analysis was used to test for Gaussian distribution of the data. Statistical significance of nominal data was assessed using chi-square tests and Fisher's exact test with a significance defined as a probability, (*p* < 0.05). Intergroup comparison was performed using the Mann-Whitney U test (no Gaussian distribution of the data) and the Kruskal-Wallis test by ranks (alpha = 0.05) to compare several subgroups. The Dunn-Bonferroni method was used for *post hoc* analysis.

### Standard Protocol Approvals, Registrations, and Patient Consents

The study has been approved by the institutional review board (IRB). Patient consent was not obtained prospectively, as the diagnostic pathway for this study was integrated into the clinical routine diagnostic procedure and was required only for patients who were contacted for follow-up investigations.

## Results

In all, 225 patients (53.8% female) with a mean age of 73 years (range: 51–94) were prospectively enrolled in the study. Seventeen (8%) of them were classified as definite or suspected AE, the remaining 208 (92%) as AE negative (cohort III).

### Cohort I: Definite AE

A total of nine patients (*n* = 9/225; 4%) met the criteria for dAE: All patients presented with well-defined neural AB in CSF and serum [anti-GAD AB (*n* = 1), anti-LGI1 AB (*n* = 1), anti-CASPR2 AB (*n* = 3), anti-NMDAR AB (*n* = 1), anti-Amphiphysin/neuropil AB (*n* = 1), anti-Hu/Sox/Zic/GABA B AB (*n* = *1*), and anti-Hu/Zic-4 AB (*n* = 1)]. Of these, three patients (*n* = 3/9) were newly diagnosed with small cell lung cancer after information about the typical onconeural AB profile (patients 7–9 in Tables 1, 2). These patients had OCB in CSF but only unilateral or no MRI signal alterations. In addition to IT and ASD treatment, they received tumor therapy according to local standards. Two patients (*n* = 2/9) had neuropsychological deficits consistent with AE (Supplementary Table 1). Neither of these patients fulfilled the clinical diagnostic criteria for definite autoimmune limbic encephalitis defined by Graus et al. (4), as they did not present with bilateral MRI brain abnormalities. Two patients (*n* = 2/9) fulfilled the Graus criteria for possible AE. Details are given in Tables 1, 2, and the Supplementary Table 1.

**Table 1:** Patients with definite AE.

**Patients with definite AE**	**Age**	**MRI**	**EEG**	**Therapy**		**Follow-up**
				**Immunotherapy**	**Anti-seizure medication**	
No. 1	87	normal	generalized continuous slow activity	methylprednisolone 1,000 mg per day i.v. for 5 days	no	lost to follow-up
No. 2	71	normal	normal	methylprednisolone 1,000 mg per day i.v., followed by prednisolone 1 mg/kg per day p.o.*	Levetiracetam 1,000 mg per day	21 months, seizure free,
No. 3	67	T2/FLAIR hyperintensity right insula, mesial temporal, temporopolar	intermittent regional slow right temporal	prednisolone 1 mg/kg per day p.o.*, azathioprine 175 mg per day	Lamotrigine 200 mg per day	37 months, seizure free,
No. 4	59	normal	normal	prednisolone 1 mg/kg per day p.o. *	Levetiracetam 2,000 mg per day	62 months, seizure free
No. 5	66	T2/FLAIR hyperintensity right temporal	normal	methylprednisolone 500 mg per day i.v. for 5 days, plasma exchange, followed by prednisolone 1 mg/kg per day p.o.*	Levetiracetam 2,000 mg per day	102 months, seizure free
No. 6	80	contrast enhancement left parietal	intermittent generalized slow with isolated ß-bursts	methylprednisolone 1,000 mg per day i.v., followed by prednisolone 1 mg/kg per day p.o.* and once cyclophosphamide 2,000 mg i.v.	valproate (dosage unknown)	lost to follow-up
No. 7	67	normal	normal	methylprednisolone 1,000 mg per day i.v. for 5 days, plasma exchange (5 cycles), prednisolone 1 mg/kg per day p.o.*	no	3 months
No. 8	61	T2/FLAIR hyperintensity right mesial temporal	normal	methylprednisolone 1,000 mg per day i.v. for 5 days, followed by prednisolone 1 mg/kg per day p.o.*	no	lost to follow-up
No. 9	67	normal	intermittent regional slow left hemisphere with isolated epileptic discharges	methylprednisolone 1,000 mg per day i.v. for 5 days, followed by prednisolone 1 mg/kg per day p.o.*	Levetiracetam 4,000 mg per day	23 months, seizure free

* *Gradual reduction in dosage over several weeks*.

*MRI, magnetic resonance imaging; EEG, electroencephalography; AE, autoimmune encephalitis; FLAIR, fluid attenuated inversion recovery; i.v., intravenous; p.o., per os*.

**Table 2:** Neural antibody and CSF results of patients with definite and suspected AE.

**Antibody (titer)**	**CSF results**				
	**Cell count/μl**	**Lactate (mmol/l)**	**Albumin quotient**	**OCB**	**Cell differentiation**
No. 1 anti-GAD65 AB (serum: 1:1280, CSF 1:8)	1	2.7	14.9	Negative	92% monocytes
					7% lymphocytes
					1% other cells
No. 2 anti-LGI1 AB [serum: 1:640, CSF 1:4 (+ anti-titin ab + unspecific neuropil ab in serum + CSF)]	2	1.7	9.6	Negative	44% monocytes 56% lymphocytes
No. 3 anti-CASPR2 AB (serum 1:2,560, CSF 1:32)	9	1.7	14.2	Negative	90% lymphocytes
					9% monocytes
					1% other cells
No. 4 anti-CASPR2 AB (serum 1:1,500,000, CSF 1:100,000)	12	2.2	7	Negative	74% monocytes
					26% lymphocytes
No. 5 anti-CASPR2 AB (serum 1:1,000, CSF 1:100)	1	1.7	5.5	Negative	65% monocytes
					32% lymphocytes
					3% other cells
No. 6 anti-NMDAR AB (serum 1:80, CSF 1:256)	11	1.5	4.4	Positive	83% lymphocytes, 16% monocytes
					1% other cells
No. 7 Amphiphysin + Neuropil AB CSF and serum	2	2	8.5	Positive	86% lymphocytes
					12% monocytes
					2% other cells
No. 8 anti-GABAbR AB serum 1:640, CSF 1:8/Hu CSF and serum; Sox/Zic CSF and serum	2	2.4	3.3	Positive	92% lymphocytes, 7% monocytes, 1% other cells
No. 9 Hu AB CSF and serum/Zic4 AB serum	14	1.7	9.2	Positive	92% lymphocytes
					3% other cells
					5% monocytes
No. 10 anti-GABABR AB serum 1:200; CSF negative	1	1.6	3.3	Negative	55% lymphocytes
					45% monocytes
No. 11 Unspecific neuropil AB in CSF and serum	9	2.9	15.6	Negative	46% lymphocytes
					1% other cells
					53% monocytes
No. 12 anti-CASPR2 AB serum 1 < 2,500, CSF negative,	70	3.6	20.8	Negative	24% lymphocytes
					75% monocytes
No. 13 AB neg	2	6.5	7.6	Negative	52% lymphocytes
					48% monocytes
No. 14 AB neg	2	3.7	5.9	Negative	74% lymphocytes
					24% monocytes
No. 15 AB neg	1	4.2	22.2	Negative	34% lymphocytes, 44% monocytes
					22% other cells
No. 16 AB neg	1	2	5.5	Negative	Not done
No. 17 unspecific neuropil AB serum, AE following HSV encephalitis	42	3.1	8.5	Positive	Not done

*CSF, cerebrospinal fluid; OCB, oligoclonal band; S, Serum; GAD, glutamic acid decarboxylase 65; LGI1, leucine-rich glioma inactivated 1 protein; CASPR-2, contactin-associated protein-like 2; NMDAR, N-methyl-D-aspartate receptor; GABA, amino-butyric acid B receptor; AB, antibody; AE, autoimmune encephalitis*.

### Cohort II: Suspected AE

Eight patients (*n* = 8/225; 3.5%) had unilateral MRI alterations suggestive for AE. Two patients (*n* = 2/8, 38%) had neuropsychological findings compatible with AE (Supplementary Table 2). CSF analysis revealed elevated cell count or OCB in CSF in four patients (*n* = 4/8; 50%). Two patients (*n* = 2/8; 25%) were suspected to have a paraneoplastic AE, one with anti-GABAB receptor AB in serum and a known B-cell non-Hodgkin's lymphoma and one with anti-CASPR2 AB in serum and a large cell bronchial carcinoma (Table 2). Applying the Graus criteria in this cohort revealed no patient with definite autoimmune limbic encephalitis, three patients (*n* = 3/8; 38%) with possible AE (2,6,7) and one patient (*n* = 1/8; 13%) fulfilled the criteria for AB-negative but probable AE (2). Details are given in Tables 2, 3 as well as in the Supplementary Table 2.

**Table 3:** Patients with suspicion of AE, not fulfilling the criteria for definite AE.

**Patients with suspected AE**	**Age**	**MRI**	**EEG**	**Therapy**		**Follow-up**
				**Immunotherapy**	**Anti-seizure medication**	
No. 1	75	T2/FLAIR hyperintensity right temporal and left parietal	normal	plasma exchange (5 cycles) followed by prednisolone 1 mg/kg per day p.o.*, azathioprine 100 mg per day	gabapentin 1,200 mg per day p.o.	12 months, seizure free, progressive psychiatric and memory decline
No. 2	61	T2/FLAIR hyperintensity right mesial temporal	normal	prednisolone 1 mg/kg per day p.o.*	levetiracetam 2,000 mg per day	lost to follow-up
No. 3	67	contrast enhancement right cingulate gyrus and mesial temporal	intermittent generalized slow and intermittent regional slow right hemisphere	methylprednisolone 1,000 mg per day i.v. for 5 days followed by prednisolone 1 mg/kg per day per os*	phenytoin 200 mg per day, valproate 1,800 mg per day	6 months, no information about seizure outcome available
No. 4	79	T2/FLAIR hyperintensity left temporal and insula	intermittent isolated epileptic discharges left frontal and temporal	prednisolone1 mg/kg per day p.o.*	brivaracetam 200 mg per day, phenobarbital 500 mg per day	lost to follow-up
No. 5	41	T2/FLAIR hyperintensity right insula	continuous generalized slow, continuous rhythmic pattern (sharp waves) over the right hemisphere	methylprednisolone 1,000 mg per day i.v. for 5 days followed by immunoadsorption, plasma exchange (5 cycles)	topiramate 150 mg per day, lacosamide 200 mg per day	14 months, seizure free
No. 6	61	T2/FLAIR hyperintensity left mesiotemporal and medial thalamus	intermittent regional slow with left temporal continuous rhythmic pattern	methylprednisolone 1,000 mg per day i.v., followed by prednisolone 1 mg/kg per day p.o.*	phenytoin 350 mg per day, brivaracetam 200 mg per day, lacosamide 400 mg per day	2 months, status epilepticus
No. 7	80	T2/FLAIR hyperintensity dorsal thalamus, mesial temporal and insula as well as bilateral occipital	continuous regional slow over the left hemisphere	Prednisolone 1mg/kg per day p.o.*	levetiracetam 3,000 mg per day, lacosamide 200 mg per day, valproate 1,800 mg per day	48 months, seizure frequency >1/year
No. 8	71	atrophy left temporopolar and temporomesial	intermittent regional slow over the left hemisphere with continuous rhythmic pattern (sharp waves), generalized slow	prednisolone 1 mg/kg per day p.o.*	levetiracetam 1,000 mg per day, valproate 1,200 mg per day	70 months, no information about seizure outcome available

* *Gradual reduction in dosage over several weeks*.

*MRI, magnetic resonance imaging; EEG, electroencephalography; AE, autoimmune encephalitis; FLAIR, fluid attenuated inversion recovery; i.v., intravenous; p.o., per os*.

### Cohort III: No Evidence of AE

This cohort comprises 208 patients (*n* = 208/225; 92%) that did not fulfill the above-mentioned diagnostic criteria for definite or suspected AE. None of the patients had specific neural AB in CSF or serum. Elevated cell count in CSF (>4/μl) was detected in 6% (*n* = 13/205) of the patients, and elevated lactate concentrations (>2.5 mmol/l) were seen in 23% (*n* = 47/207) of the patients and elevated concentrations of total protein (>500 mg/dl) in 52% (*n* = 108/207) of the patients. OCB in CSF were present in 4.5% (*n* = 8/179) of the patients. Most of these patients had a cryptogenic origin of seizures (*n* = 121/208, 58%), 84 patients had a symptomatic origin (*n* = 84/208, 40%), two patients an idiopathic (*n* = 2/208, 1%) origin, and one patient had an acute symptomatic seizure. MRI analysis revealed no changes indicative of AE in 86% (*n* = 134/155) of patients. Twelve patients (*n* = 12/155; 8%) presented with mesial temporal unilateral volume changes and nine patients (= 9/155; 6%) demonstrated unspecific extratemporal T2/FLAIR hyperintensities. Over half of this cohort (*n* = 106/208, 51%) received neuropsychological testing.

### When Should We Suspect AE in Late-Onset Seizures?

Compared with patients with no evidence of AE, those with definite and suspected AE *(dAE/sAE)* (i) were significantly younger (*p* = 0.028), (ii) more frequently presented with a history of/or an active tumor disease (*p* = 0.006), (iii) showed specific mesial temporal neuropsychological deficits (*p* = 0.0012), (iv) more frequently elevated CSF cell counts >4/μl (*p* = 0.0002) and isolated OCB (*p* = 0.0012) in CSF, and (v) unilateral mesial temporal T2-hyperintensities in MRI (*p* = 0.0001).

Intergroup comparison between *dAE/sAE* and *nAE* revealed no significant differences concerning mRS (*p* = 0.16), frequency of abnormal EEG patterns, semiology (*p* = 0.06), motor vs. non-motor seizure onset (*p* = 0.13), and occurrence of status epilepticus (*p* = 0.5) (for more detail see Table 4).

**Table 4:** Comparison of clinical characteristics between AE cohorts and cohort III.

	**seizures due to AE *n* = 17**	**seizures due to another etiology *n* = 208**	*p*-value*
Age	67 (61;75)	75 (66;80)	0.028*
Female (*n*;%)	8;47	113;54	0.62
mRS	2 (0;4)	0 (0;3)	0.162
Status epilepticus (*n*;%)	4;24	33;15.9	0.5
Semiology			0.06
FIAS (*n*;%)	8;47	120;58	
FAS (*n*;%)	4;24	15;7.2	
GMS (*n*;%)	2;12	59;28	
≥2 (*n*;%)	1;6	9;4.3	
Onset (motor) (*n*;%)	5;29	114; 55	0.13
Malignancy (active or known) (*n*;%)	8;47	35; 16.9	0.006*
EEG			
ED (*n*;%)	6; 35;	51;25.4	0.393
Generalized slowing (*n*;%)	4; 24	63;31	0.594
Regional slowing and/or amplitude decrease (*n*;%)	6;35	44;22	0.2311
Neuropsychological impairment			
Mesial temporal^‡^ (*n*;%)	4; 36	3; 3	0.0012
No cognitive impairment (*n*;%)	3;27	27;25	>0.999
CSF			
CC (> 4/μl) (*n*;%)	7; 41	13;6	0.0002*
Lactate (> 2.5 mmol/l) (*n*;%)	8;47 (1.7;3.1)	47;23	0.0374
Total protein (> 500 mg/dl)	11;65	108;52	0.45
OCB pos (*n*;%)	5;29	8;4.5	0.0012*
MRI^†^			<0.00001*
No lesion (*n*;%)	5;56	134;86	0.0315
Unilateral lesion (*n*;%)	10;59	12; 8	0.0001
Bilateral lesion (*n*;%)	0;0	0;0	
Extratemporal lesion (*n*;%)	1; 6	9;6	>0.99

*Nominal data are given as percentages, and continuous data are expressed as the median (1st; 3rd quartile)*.

* *Significance: p-value ≤ 0.05; MRI: epileptogenic lesions of unknown origin, possible autoimmune (T2/FLAIR-hyperintensity and/or swelling and/or Gd enhancement), Neuropsychological assessment compatible with deficits in mesial temporal structures: amnestic syndrome and/or delayed verbal memory and/or impaired executive functioning*.

*Mrs, modified Rankin Scale; EEG, electroencephalography; ED, epileptic discharges; MRI, magnet resonance imaging; Gd, gadolinium; CSF, cerebrospinal fluid; CC, cell count; OCB, oligoclonal band; FIAS, focal onset impaired awareness seizure; FAS, focal onset awareness seizure; GMS, generalized motor seizure*.

### Treatment and Outcomes

**Figure 1:**
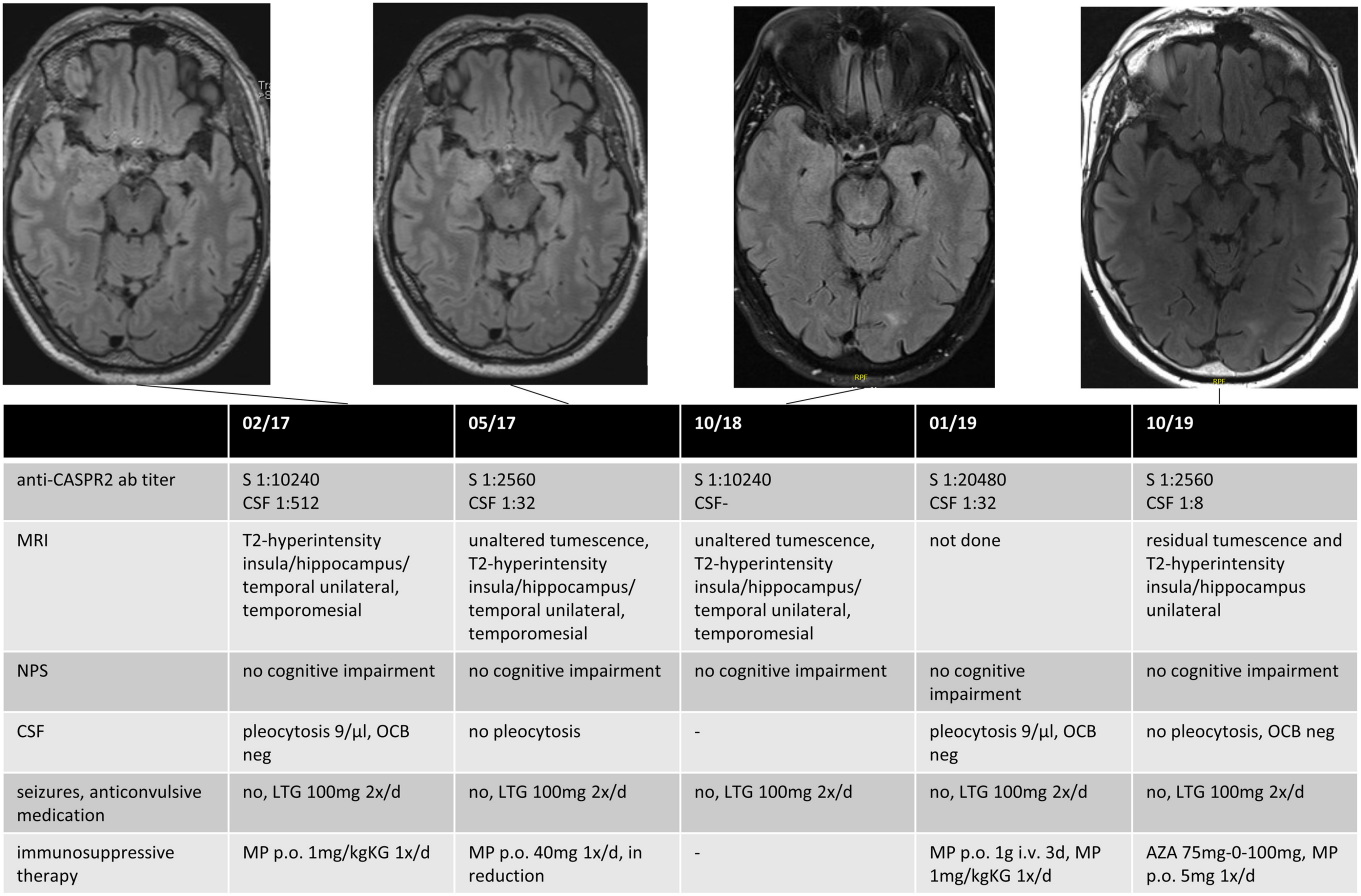
Data for an anti-CASPR2 AB positive patient. The patient had a first epileptic seizure (GMS) 3 months before the diagnosis and recurrent focal seizures until he received monotherapy with Lamotrigin, after which he became seizure-free. The first MRI showed unilateral swelling and T2-hyperintensity in the insula, the hippocampus, temporal mesial, and in the temporal lobe, compatible with possible AE, although not fulfilling the Graus criteria in the absence of neurocognitive or neuropsychiatric deficits. Immunotherapy was initialized after positive testing for anti-CASPR2 AB in serum and CSF. The patient had no clinical signs of AE until last FU; immunotherapy was reinitialized 2 years after diagnosis because of an increased titer of anti-CASPR2 AB in routine clinical FU and a recurrent pleocytosis in CSF. Anti-CASPR2 contactin-associated protein-like 2, GMS generalized motor seizure, MRI magnet resonance imaging, AE autoimmune encephalitis, CSF cerebrospinal fluid, FU follow-up, S Serum, NPS neuropsychological testing, OCB oligoclonal bands, LTG Lamotrigin, MP Methylprednisolon, AZA Azathioprin.

A follow-up (FU) was carried out in 59% (*n* = 10/17) of patients with *dAE* or *sAE*, with a mean FU time of 40 months (range: 6–102) after the first seizure disorder. Of the remaining seven patients, five (29%) were lost to follow-up (three patients with dAE, two patients with sAE), and two had a follow-up of <6 months. Among those with FU, all were seizure-free at last FU, three of them with the first used ASD. First-line IT was administered to all patients. Second-line IT was needed in one patient with anti-CASPR2 AB encephalitis due to newly manifested CSF pleocytosis and increasing serum AB titers over the course of the disease (Figure 1). One patient with sAE received a second-line IT due to progressive psychiatric and neuropsychological deficits (Table 3).

**Figure 2:**
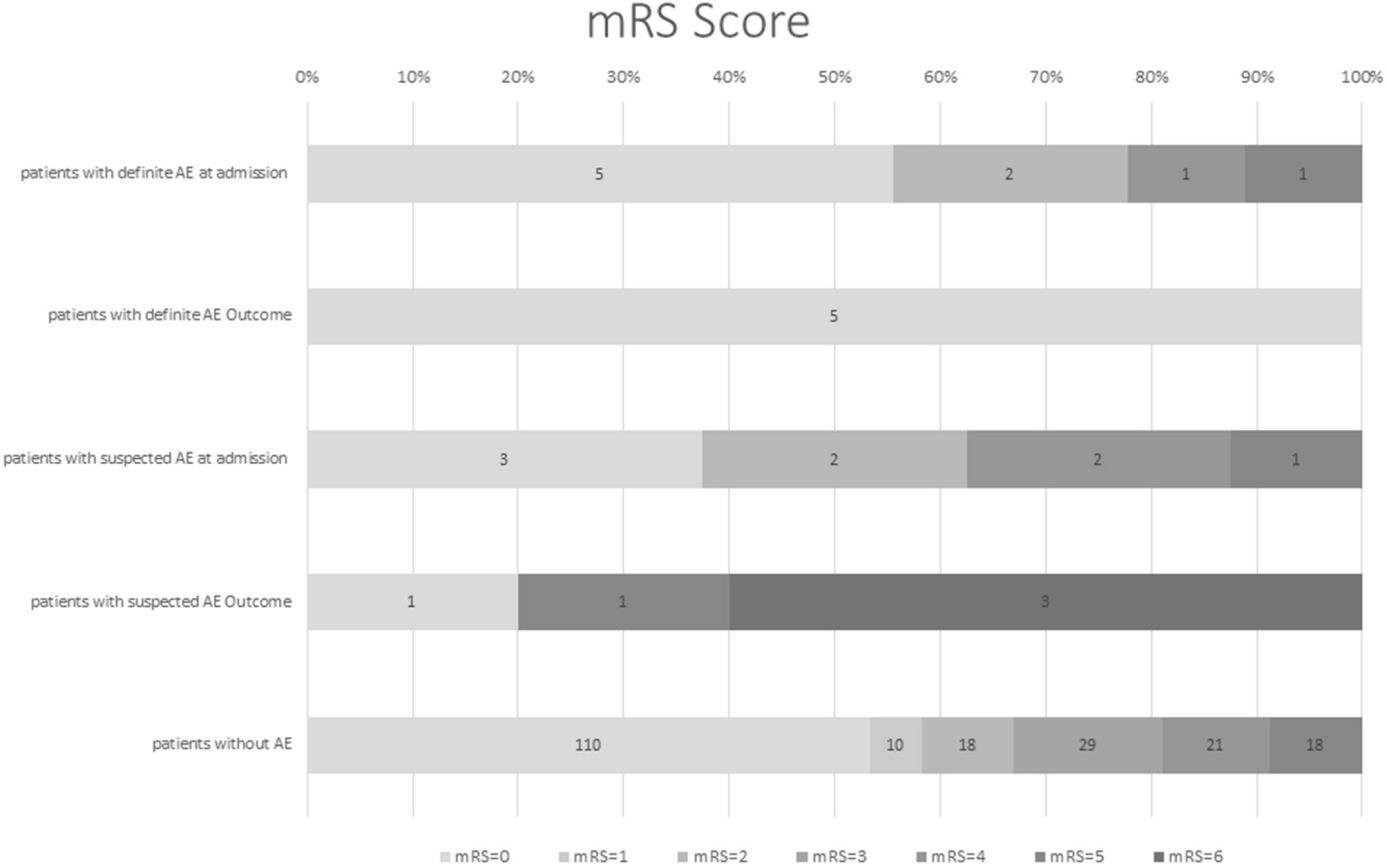
Modified Rankin scale score (mRS) of patients with definite AE and suspected AE at first hospital admission and FU in comparison with patients with late-onset seizures due to another etiology. Modified Rankin scale at hospital admission was low for patients with definite and suspected AE (71% with mRS ≤2), which decreased to 60% with mRS ≤2 at last FU [mean follow-up time of 40 months (range 6–102)]. Outcome was favorable for non-paraneoplastic and definite AE; outcome in paraneoplastic and suspected AE was worse. However, the generalizability of these study results is limited by the small number of patients. mRS, modified Rankin scale, AE, autoimmune encephalitis, FU, follow-up.

At hospital admission mRS was low (≤2) in the majority of *dAE*/*sAE* patients (*n* = 12/17, 71%), decreasing to a proportion of 60% (*n* = 6/10) at last FU (Figure 2). Outcome was favorable for non-paraneoplastic AE (mRS = 0 for all patients with anti-CASPR2 AB and the one patient with anti-LGI1 AB); outcomes in paraneoplastic AE and suspected AE, however, were worse (mRS = 0 in only two patients). Five of 17 patients (29%) with *dAE*/*sAE* died during FU, and after evaluation of the medical charts, three of them (60%) had paraneoplastic AE. One patient died because of infection probably associated to IT, another patient died of a comorbid disease. Of the five patients with paraneoplastic AE, three patients (60%) died.

## Discussion

In our cohort, patients with definite and suspected AE comprised 8% of the study population. Definite AE was found in 4% of all patients: all of them had AB in serum and CSF. To our knowledge, this is the largest study aimed at investigating the prevalence and clinical features of AE with late-onset seizures as the first clinical symptom.

Overall information about specific age-related differences of clinical features, treatment, and outcomes of AE is scarce and prospective studies to elucidate this topic are lacking. There is only limited information about AB prevalence in elderly patients. In a patient cohort >60 years of age with no signs of inflammation, the most common antibodies detected were anti-LGI1 AB (31.4%) and anti-IGLON5 AB (28.6%), whereas, anti-NMDAR AB and anti-CASPR2 AB are less common (17). Overall, the most common antibodies >60 years are anti-LGI1 AB (34%), followed by anti-GABABR AB (17%), anti-AMPAR AB (13%), anti-NMDAR AB (11%), anti-IGLON5 AB (9%), and anti-CASPR2 AB (8%) (17). Older patients with AE have a higher risk of having a malignancy (22), but this depends on the subtype of the AE (23).

### When to Suspect AE in Patients With Late-Onset Seizures

Consistent with a previous study showing that 23% of patients aged >60 years with positive AB had no signs of inflammation in their diagnostic work-up (17), 12% of our patients presented without inflammatory changes in both MRI and CSF.

Magnetic resonance imaging shows that patients with AE as the cause of late-onset seizures often present with unilateral alterations in mesial temporal structures. In our cohort this was the case in 71% of patients. However, although unilateral MRI changes are common in AE, they are a limiting factor for fulfilling the diagnostic criteria proposed by Graus et al. (4, 11), at least in the absence of neural AB. As unilateral mesial temporal MRI changes are not specific for AE and may also occur in non-autoimmune disorders, such as lower grade gliomas or hippocampal sclerosis, they are not a criterion for definite AE (3, 4). Consequently, unilateral MRI alterations should always be interpreted in the context of other clinical features of AE.

Patients with dAE or sAE more frequently showed a pleocytosis and/or OCB in CSF than patients without AE, which is a frequent finding in patients with AE (4, 24). The reported percentages of pathological values for the three basic CSF parameters (cell count, protein, OCB) are highly different among the AB-defined subtypes of AE (25). There are no specific age-focused studies regarding CSF changes in patients with AE. In the study by Blinder et al. (25), the median age of patients with anti-LGI1 AB, anti-CASPR2 AB, anti-GABABR AB, anti-IGLON5 AB, and anti-AMPAR AB was over 60 years old (22). Thus, it can be concluded that older patients with anti-LGI1 AB encephalitis less frequently have OCB in CSF, whereas, patients with anti-CASPR2 AB encephalitis more frequently show higher cell counts (29%−36%) as well as OCB (23%−32%) in CSF (22). All in all, inflammatory CSF changes are more often found in NMDA, GABAB, and AMPA receptors, as well as DPPX in contrast to patients with CASPR2, LGI1, GABAA, or glycine receptor AB, with mostly normal CSF findings (25). Nevertheless, we observed a significant proportion of patients with pleocytosis and/or OCB in CSF in our study, which may be indicative of AE even in older patients with different subtypes of AE. Although other studies recommend CSF analysis only to rule out infections (8, 26), our data suggest performing AB tests if the CSF cell count is increased or OCB are detected exclusively in CSF.

Except for faciobrachial dystonic seizures (FBDS) in anti-LGI1 AB encephalitis, no specific semiological features are known to be pathognomonic for AE, even in cases of status epilepticus.

Although not confirmed by our data, an EEG often provides important ways to suspect AE (8); however, EEG interpretation strongly depends on the rater's experience to identify characteristics of AE, such as a delta brush pattern in anti-NMDAR AB encephalitis (18).

One of the most common neural AB detected in this elderly population was anti-CASPR2 AB. Only one of our patients (aged 80 years) presented with anti-NMDAR AB, which is usually found in young females with AE and only rarely (5–12%) in the elderly population (15, 27, 28). Interestingly, in contrast to the younger population with anti-NMDAR AB encephalitis, elderly patients are more often male and show a milder course of the disease. However, the long-term outcome is worse due to age-related factors and the higher risk of delayed diagnosis (15). Even though the outcome is worse in comparison with younger patients, a significant proportion of patients fully recover after the acute phase of the disease (15). Prior studies have reported a frequent occurrence of anti-CASPR2 AB in elderly patients (with a median age of 60) with AE (18), which is supported by our results. Consistent with our findings (mRS ≤2 in 71% of patients at hospital admission), the clinical appearance of anti-CASPR2 AB encephalitis is rather mild (29). Additionally, MRI changes in anti-CASPR2 AB encephalitis may evolve over a long period (29) and are, therefore, found in only about 40% at the clinical disease onset (18). It is quite conceivable that early initiation of IT prevented worse clinical outcomes in our patients and contributed to the mild appearance of clinical features and the rather minor MRI alterations.

Escudero et al. (17) reported that rapidly progressive dementia was not a frequent clinical presentation of late-onset autoimmune encephalitis. Our results show that specific mesial temporal neuropsychological deficits were more frequent in patients with dAE/sAE compared with those without AE. Among patients with late-onset seizures in whom AE is at least suspected, neuropsychological testing should therefore focus on mesial temporal deficits and should be repeated during the course of the disease. Consistent with the circumscribed structural alterations of mesolimbic structures, characteristic neuropsychological deficits in AE mainly comprise impairments of episodic memory (material-specific, i.e., verbal and/or non-verbal, according to the lateralization of the lesion) and correlate with disease severity, the extent of structural (especially mesial temporal) alterations, and antibody titer and the time at which an immunotherapy is started (15, 28). Other tests comprise specific tests for figural (e.g., ROCFT, BVMT) and verbal memory (e.g., RAVLT/VLMT) that are proven to be sensitive for mesial temporal functions in patients with epilepsy (30) and especially those with in AE (31) and should be included in the test protocol (32). Especially in the higher-aged population, short dementia screenings as MMST (Mini Mental Status Test) or MoCA (Montreal Cognitive Assessment) are not adequate tools because of their low resolution to differentiate memory processes (learning, recall, recognition) and to contrast different cognitive domains. Instead, a comprehensive neuropsychological test battery focusing on verbal and figural memory but including other domains, such as attention/cognitive speed, executive functions, and language functions should be performed in order to weight the memory deficits against other cognitive functions (interpretation of the neuropsychological profile).

When we applied the clinical diagnostic criteria from Graus et al. (4) to our patient cohort, no patient with either definite AE or suspected AE fulfilled the criteria for definite autoimmune limbic encephalitis. This is highly relevant, as these patients would have been missed for AB testing in the acute phase. This is likely due to the specific AB prevalence in this patient cohort. As many patients have either anti-CASPR2 AB or onconeural AB without typical MRI features for definite limbic encephalitis as described above, they are missed when researchers apply the Graus criteria. Newer prognostic pathways, such as the APE or RITE Score (24) may be more precise in these patients but were not yet established at the time of conception of our study.

### Treatment and Outcome

Late-onset seizures in non-paraneoplastic AE are known to have a favorable prognosis (3, 18), corresponding to our findings with a high seizure-free rate with at least one ASD and/or IT. Overall impairment at hospital admission was low (47% mRS 0, 71% mRS ≤2), probably due to the high rate of anti-CASPR2 AB AE (24%) in our patient cohort (25). Among patients with paraneoplastic AE, 60% died, mostly due to the underlying malignancy. Other factors may influence outcome parameters in our patient cohort, such as limited capacity of recovery after brain damage in the aging brain (33). Furthermore, age itself is considered an independent risk factor for adverse outcomes in late-onset anti-NMDAR encephalitis (15).

Although randomized controlled trials on IT in AE are lacking, about 70% of AE patients respond to gradual IT escalation (18) including those with paraneoplastic AE (34). First-line therapy comprises corticosteroids, intravenous immunoglobulins, and/or PEX/ (IA) (18, 35). Early administration of high-dose intravenous corticosteroids is associated with improved clinical outcome (18). All patients in our cohort received high-dose corticosteroids as initial therapy (administered orally or intravenously), followed by a gradual reduction in dosage in 88% of patients. Only three patients received a second-line immunotherapy. One of them developed a severe infection and died during the course of the disease. In view of the high rate of multimorbidity and polypharmacy in this patient population as well as the often favorable outcome among patients with non-paraneoplastic AE patients under first-line therapy (36), administration of second-line IT should be considered with caution. This recommendation is supported by a study among patients with multiple sclerosis that found a risk of adverse effects of immunotherapy (such as opportunistic infections and malignancies) that increases with the patient's age (37). Published immunotherapy protocols include patients aged up to 85 years; younger patients are, however, clearly overrepresented in these studies (15, 38)). Randomized controlled trials based on standard immunotherapy protocols including elderly patients with autoimmune encephalitis are lacking (38).

As most non-paraneoplastic AE patients have a favorable outcome, we suggest using second-line IT only in cases of recurrent disease attacks verified by MRI and/or CSF inflammation.

### Limitations

One major limitation of the study is the single-institutional design, which leads to a limited number of AE cases, further limiting statistical assertions and the possibility of comparing different subgroups (dAE and sAE). In addition, not all patients with AE received an FU investigation as this was not part of the initial study protocol.

## Conclusion

To date, there are only limited data available concerning diagnostic strategies, clinical symptoms, treatment, and outcomes of autoimmune encephalitis in the elderly. The results of our study provide clinicians with additional information verified in a great cohort of 225 patients on the prevalence and outcomes of AE as well as predictors of when to suspect AE in patients with late-onset seizures. Although characteristic signs of inflammation in AE may be lacking especially in elderly patients, the presence of CSF and MRI signs of inflammation, mesial temporal neuropsychological alterations, younger age, a known malignancy and specific semiological features (such as FBDS) should suggest AE. An epileptic seizure may be the first symptom of AE. In short, AB testing in CSF and sera, cerebral MRI, lumbar puncture, and neuropsychological testing for mesial temporal deficits should be part of the diagnostic protocol for AE following late onset seizures. First-line therapy comprises ASD as well as corticosteroids and/or PEX/IA; in view of the potential comorbidity and polypharmacy in the elderly, administration of second-line IT should be considered with caution.

## Data Availability Statement

The raw data supporting the conclusions of this article will be made available by the authors, without undue reservation.

## Ethics Statement

The studies involving human participants were reviewed and approved by Institutional review board of the university medicine Greifswald, Germany. Written informed consent for participation was not required for this study in accordance with the national legislation and the institutional requirements.

## Author Contributions

MS analyzed the data, interpreted the data, drafted the manuscript, and revised the manuscript for intellectual content. MZ had a major role in the acquisition and analysis of the data. VP revised the manuscript for intellectual content and interpreted the data. FP revised the manuscript for intellectual content and design and conceptualized the study. All authors contributed to the article and approved the submitted version.

## Conflict of Interest

MS reports personal fees and grants from Merck Healthcare Deutschland and Bayer Vital GmbH. FP obtained honoraria for speaking engagements from Desitin Pharma (Hamburg, Germany), EISAI Pharma (Frankfurt am Main, Germany), UCB Pharma (Monheim, Germany), BIAL (Mörfelden-Walldorf, Germany), and GW Pharma (München, Germany) and was part of the speakers bureau of Desitin Pharma (Hamburg, Germany), EISAI Pharma (Frankfurt am Main, Germany), UCB Pharma (Monheim, Germany), BIAL (Mörfelden-Walldorf, Germany), and GW Pharma (München, Germany). The remaining authors declare that the research was conducted in the absence of any commercial or financial relationships that could be construed as a potential conflict of interest.
