# Supplementary material for: Autoimmune Encephalitis in Late-Onset Seizures: When to Suspect and How to Treat
Source: Front Neurol. 2021 Apr 7;12:633999. doi: 10.3389/fneur.2021.633999 (PMC8058403; doi:10.3389/fneur.2021.633999)
Supplement: Supplementary file 1 [file Table_1.DOCX]

Supplementary Material

1. **Determination of neural AB in CSF and serum** (performed by the MVZ Labor Krone GbR, Siemensstraße 40, 32105 Bad Salzuflen, Germany): The following methods were used: (1) indirect immunofluorescence on mouse brain for neuropil Abs and GAD65-Abs (sagittal mouse sections provided by Euroimmun, Luebeck, Germany); (2) cell-based assays for Abs against LGI1, CASPR2, NMDAR, GlyR, AMPAR1/2, GABABR, GAD65, DPPX, and mGluR5 (biochips with transfected and slightly fixed cells produced by Euroimmun; serum was diluted to 1:16, CSF was used undiluted; incubation time 30 min at room temperature [RT]; secondary antibody: goat-anti-human immunoglobulin G [IgG; heavy and light chain [H+L]) conjugated with Alexa Fluor 594 (Jackson ImmunoResearch, West Grove, PA, U.S.A., Code No. 109-585-088), dilution 1:100, incubation 30 min at RT. IgG positivity was confirmed by goat-anti-human antibody against the Fcc fragment of IgG (conjugation and manufacturer as with H+L antibody, catalog no. 304-585-008) at a dilution of 1:100, incubation 30 min at RT. Experienced readers determined if an Ab was present using the signal of the surrounding (supposedly negative) fields as respective negative controls. Abs to surface antigens were endpoint-titrated in steps that are multiples of 1:2. In addition, all samples were tested by indirect immunofluorescence on mouse brain sections and immunoblots (Ravo, Freiburg, Germany) for onconeural antibodies. Onconeural antibodies were diagnosed if both tests gave congruent results.

**Supplementary Table 1: Neuropsychological assessment of patients with definite AE**

| subject | edy y | attention/cognitive speed | memory^2^ | executive functions | language functions | assessment |
| --- | --- | --- | --- | --- | --- | --- |
| #1 | 87 |  |  |  |  | not feasible^1^ |
| #2 | 71 | average | average | average | (above) average | CERADplusNTB |
| #3 | 67 | average | average | average |  | CERADplusNTB, WAIS-IV ZN, MWT-B, LPS 3 |
| #4 | 59 | average | average | average | n/a | TMT, CVLT-K, LT, WAIS-IV ZN, ROCFT, FWIT |
| #5 | 65 | very poor | below average | below average | average | TMT, ZVT, WMS-R, VLMT, VAT, MWT-B, LPS 3 |
| #6 | 80 |  |  |  |  | not feasible^1^ |
| #7 | 67 |  |  |  |  | not feasible^1^ |
| #8 | 61 | below average | very poor | below average | below average | CERADplusNTB |
| #9 | 67 | average | average | (below) average | (below) average | CERADplusNTB |

Subject’s performance in 1^st^ neuropsychological assessment. Above average: performance >1 and ≤2 SD above mean score of normal population; average: performance in between ±1 SD of mean score of normal population; below average: performance >1 and ≤2 SD below mean score of normal population; very poor: performance >2 SD below mean score of normal population

^1^ Standardized assessment not feasible (e. g. due to aphasia or insufficient cognitive capacity)

^2^ if not otherwise stated no significant difference between verbal and figural memory performance

n/a: not assessed

edu y: number of education years; CDT: Clock Drawing Test; CERADplusNTB: Consortium to Establish a Registry for Alzheimer’s Disease: neuropsychological test battery; CVLT-K: California Verbal Learning Test, Short Form; FWIT: Stroop Test [Farbe-Wort-Interferenztest]; LPS 3: non-verbal intelligence, subtest 3 [Leistungsprüfsystem]; LT: Labyrinth Test (Chapuis) [Labyrinthtest]; MWT-B: verbal intelligence [Mehrfachwahlwortschatztest]; NAI ZST: Nürnberger Altersinventar: Subtest Digit-Symbol-Task [Zahlen-Symbol-Test]; ROCFT: Rey-Osterrieth Complex Figure Test; TMT: Trail Making Test; VAT: Visual Association Task [Visueller Assoziationstest]; VLMT: Verbal Learning and Memory Test [Verbaler Lern- und Merkfähigkeitstest]; WAIS-IV ZN: Wechsler Adult Intelligence Scale (Fourth Edition): subtest Digit Span; ZVT: Trail Making Task [Zahlen-Verbindungs-Test], AE autoimmune encephalitis

**Supplementary Table 2: Neuropsychological assessment of patients with suspected AE**

| subject | edu y | attention/cognitive speed | memory^2^ | executive functions | language functions | assessment |
| --- | --- | --- | --- | --- | --- | --- |
| #1 | 10 | n/a | very poor | very poor | average | possibly confounded with previous cerebral injury and psychological state  CERADplusNTB: subtests naming, constructional praxis; CVLT-K; WAIS-IV ZN |
| #2 | 7 | very poor | verbal: average  non-verbal: below average | very poor | average | CERADplusNTB, NAI: subtests FWT, LT, ZST; AAT TT |
| #3 | n/a |  |  |  |  | not feasible^1^ |
| #4 |  |  |  |  |  | not feasible^1^ |
| #5 | 12 | very poor | verbal: very poor  non-verbal: average | below average | n/a | TAP: subtests AL, GNG; HAWIE-R ZN; BTT; RBMT; VOSP subtest 8 |
| #6 | 16 | average | verbal: below average  non-verbal n/a | (above) average | n/a | WAIS-IV ZN, CVLT-S, BADS RS, FWIT, TMT |
| #7 | 11 | below average | verbal: very poor  non-verbal: below average | n/a | very poor | assessment confounded by aphasia  CERADplusNTB, NET subtest 3 |
| #8 |  |  |  |  |  | not feasible^1^ |

Subject’s performance in 1^st^ neuropsychological assessment. Above average: performance >1 and ≤2 SD above mean score of normal population; average: performance in between ±1 SD of mean score of normal population; below average: performance >1 and ≤2 SD below mean score of normal population; very poor: performance >2 SD below mean score of normal population

^1^ Standardized assessment not feasible (e. g. due to aphasia or insufficient cognitive capacity)

^2^ if not otherwise stated no significant difference between verbal and figural memory performance

n/a: not assessed

edu y: number of education years; AAT: Aachener Aphasie Test: subtest Token Test; BADS RS: Behavioural Assessment of the Dysexecutive Syndrome, subtest Rule Shift; BTT: Block Tapping Test; CERADplusNTB: Consortium to Establish a Registry for Alzheimer’s Disease: neuropsychological test battery; CVLT-S: California Verbal Learning Test, Standard Form; CVLT-K: California Verbal Learning Test, Short Form; FWIT: Stroop Test [Farbe-Wort-Interferenztest]; HAWIE-R: Hamburg-Wechsler-Intelligenztest (Revised) [Wechsler Adult Intelligence], subtest Digit Span; NAI: Nürnberger Altersinventar: subtest FWT: Stroop Test [Farb-Wort-Test], LT Labyrinth Test, ZST Digit-Symbol-Task [Zahlen-Symbol-Test]; NET: Neglect-Test, subtests: 1 (Line Cancellation), 3 (star cancellation), 4-6 (copy tasks), 7 (line bisection); RBMT: Rivermead Behavioural Memory Test, subtests Picture Recognition [Bilder], Remembering a newspaper article [Geschichte]; Tagesplanungsaufgabe: Planning Task; TAP: Tests of Attentional Performance, subtests: AL (Alertness), GNG (Go/Nogo); TMT: Trail Making Test; VOSP: Visual Object and Space Perception Battery, subtest 8 Cube Analysis; WAIS-IV ZN: Wechsler Adult Intelligence Scale (Fourth Edition): subtest Digit Span; ZVT: Trail Making Task [Zahlen-Verbindungs-Test], AE autoimmune encephalitis
